# Rubicon in Metabolic Diseases and Ageing

**DOI:** 10.3389/fcell.2021.816829

**Published:** 2022-01-10

**Authors:** Satoshi Minami, Shuhei Nakamura, Tamotsu Yoshimori

**Affiliations:** ^1^ Department of Genetics, Graduate School of Medicine, Osaka University, Suita, Japan; ^2^ Department of Intracellular Membrane Dynamics, Graduate School of Frontier Biosciences, Osaka University, Suita, Japan; ^3^ Institute for Advanced Co-Creation Studies, Osaka University, Suita, Japan; ^4^ Integrated Frontier Research for Medical Science Division, Institute for Open and Transdisciplinary Research Initiatives, Osaka University, Suita, Japan

**Keywords:** Rubicon, autophagy, LAP, metabolic disease, ageing, longevity

## Abstract

Autophagy is a conserved cellular degradation system that maintains intracellular homeostasis. Cytoplasmic components are engulfed into double-membrane vesicles called autophagosomes, which fuse with lysosomes, and resulting in the degradation of sequestered materials. Recently, a close association between autophagy and the pathogenesis of metabolic diseases and ageing has become apparent: autophagy is dysregulated during metabolic diseases and ageing; dysregulation of autophagy is intimately associated with the pathophysiology. Rubicon (Run domain Beclin-1 interacting and cysteine-rich containing protein) has been identified as a Beclin-1 associated protein. Notably, Rubicon is one of few negative regulators of autophagy whereas many autophagy-related genes are positive regulators of autophagy. Rubicon also has autophagy-independent functions including phagocytosis and endocytosis. In this mini-review, we focus on the various roles of Rubicon in different organs in the settings of metabolic diseases and ageing, and discuss its potential role as a promising therapeutic target.

## Introduction

The prevalence of metabolic diseases and age-related diseases is significantly increasing in the world (Christensen et al., 2009; Mozumdar and Liguori, 2011; von Ruesten et al., 2011; Ranasinghe et al., 2017; Foreman et al., 2018). Therefore, the development of effective treatment against these diseases is a pressing issue. Autophagy is a cellular degradation system that maintains intracellular homeostasis (Levine and Kroemer, 2008; Mizushima et al., 2008). Accumulating evidence supports that autophagy protects against metabolic diseases (Zhang et al., 2018) and age-related diseases (Hansen et al., 2018). In 2009, Rubicon (Run domain Beclin-1 interacting and cysteine-rich containing protein) has been discovered as a protein that negatively regulates autophagy (Matsunaga et al., 2009b; Zhong et al., 2009). Subsequent researches have clarified that Rubicon has various functions besides autophagy, and is intimately related to the pathogenesis of metabolic diseases and age-related diseases. In this mini-review, we first outline the diverse molecular functions of Rubicon. Next, we summarize the recent findings regarding the role of Rubicon in metabolic diseases and age-related diseases. Finally, we discuss current issues and propose future directions for the therapeutic application of Rubicon.

## The Molecular Function of Rubicon

**FIGURE 1:**
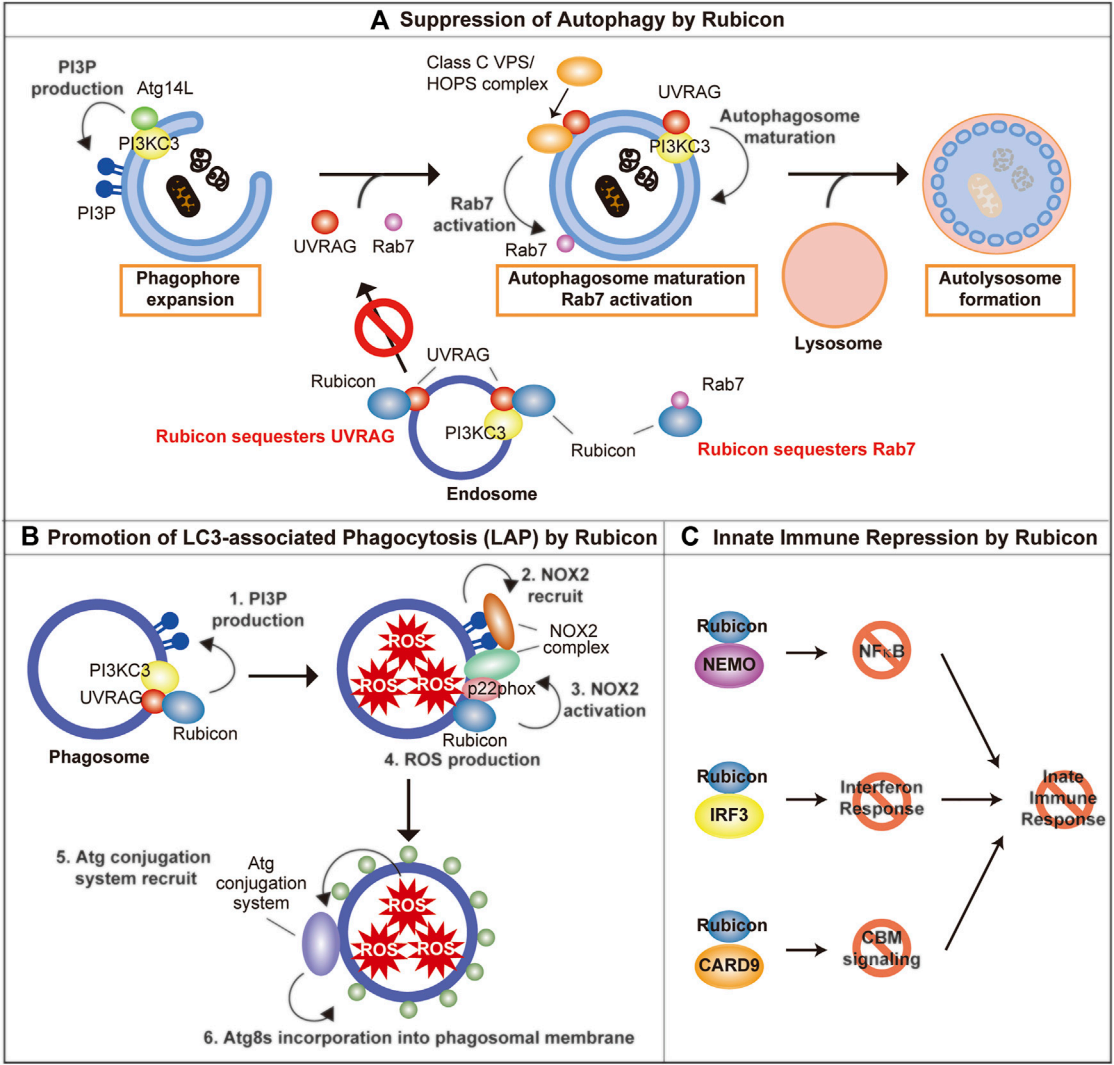
The molecular function of Rubicon. **(A)** Rubicon suppresses autophagosome maturation via the sequestration of UVRAG, PI3KC3, and Rab7. **(B)** Rubicon promotes LAP *via* promotion of PI3P production and stabilization of NOX2 complex on phagosomes. **(C)** Rubicon inhibits innate immune response via the sequestration of NEMO, IRF3, and CARD9. PI3P, phosphatidylinositol-3-phosphate; PI3KC3, Class III phosphatidylinositol-3-kinase; NEMO, NF-κB essential modulator; IRF3, interferon regulatory factor 3; CARD9, caspase recruitment domain-containing protein 9.

Rubicon has been discovered as a negative regulator of autophagy in 2009 (Matsunaga et al., 2009b; Zhong et al., 2009). Besides autophagy, Rubicon has important roles in phagocytosis, endocytosis, microbial infection and innate immune response. In this session, we summarize the autophagy-dependent and–independent function of Rubicon (Figure 1).

### Autophagy-Dependent Function of Rubicon

Class III phosphatidylinositol-3-kinase (PI3KC3), which consists of three core components, Vps34, p150, and Beclin-1 (Simonsen and Tooze, 2009; Sun et al., 2009), plays an important role for autophagosome biogenesis by catalyzing the formation of phosphatidylinositol-3-phosphate (PI3P) (Simonsen and Tooze, 2009). The core PI3KC3 complex interacts with mutually exclusive molecules Atg14L and UVRAG (Liang et al., 2006; Itakura et al., 2008; Sun et al., 2008). Atg14L contributes to phagophore expansion which leads to autophagosome formation, while UVRAG plays an important role in autophagosomal maturation (Liang et al., 2006). UVRAG is also reported to activate Rab7 by interacting with class C Vps/HOPS complex, which leads to promote the fusion between autophagosome, and lysosome (Liang et al., 2008).

Rubicon directly binds with UVRAG and Vps34 (one of PI3KC3 complex components), and suppresses their function, which impedes the maturation of autophagosome (Matsunaga et al., 2009b; Sun et al., 2011; Zambrano et al., 2019). In addition, Rubicon sequesters UVRAG from class C Vps/HOPS complex, which inhibits the activation of Rab7 (Sun et al., 2010). Rubicon also suppresses autophagic activity by directly interacting Rab7 via its RH domain (Bhargava et al., 2020). The function of Rubicon is regulated by its phosphorylation: The protein kinase HUNK-mediated phosphorylation of Rubicon inhibits its function and promotes autophagy (Zambrano et al., 2019).

Intriguingly, knockdown of Rubicon also promotes autophagosome formation (Matsunaga et al., 2009b), suggesting that the regulation of autophagosome maturation by Rubicon could be the rate limiting step for autophagic activity (Matsunaga et al., 2009a). Future research will be needed to elucidate the molecular mechanism regarding the regulation of autophagosomal formation by Rubicon.

### Autophagy-Independent Function of Rubicon

#### LAP and LANDO

Recently, several components of autophagic machinery are closely associated with vesicular trafficking, including phagocytosis and endocytosis (Galluzzi and Green, 2019). Atg8s are reported to be incorporated into various degradative single-membrane vesicles like phagosome and endosome, and Atg8s help the smooth degradation of these vesicles in lysosomes. The incorporation of Atg8s into phagosomes and endosomes are called LC3-associated phagocytosis (LAP) (Romao and Münz, 2014) and LC3-associated endocytosis (LANDO) (Heckmann et al., 2019), respectively. Interestingly, the formation of LAP and LANDO is dependent on ATG8s-conjugation system, however, independent on autophagy-initiation machinery (Nieto-Torres et al., 2021). LAP assists efficient degradation of phagocytosed pathogens and cellular debris via acidification and maturation of phagosome as well as NOX2-dependent ROS production within phagosomes (Martinez et al., 2011). Therefore, LAP deficiency exacerbates infection via insufficient degradation of pathogens, and closely correlates with excessive activation of immune cells via insufficient degradation of cellular debris, and which lead to autoimmune diseases (Martinez et al., 2016; Heckmann et al., 2017). Furthermore, LAP in macrophage induces immune tolerance under the tumor environment via promoting polarization toward M2 phenotype (Cunha et al., 2018). LANDO is reported to be closely associated with endocytic receptor recycling (Heckmann et al., 2019). Interestingly, whereas Rubicon is a negative regulator of autophagy, Rubicon is reported to be an indispensable molecule of LAP and LANDO (Martinez et al., 2015; Martinez et al., 2016; Heckmann et al., 2019). The molecular mechanism of Rubicon to regulate LAP is following: 1) Rubicon recruits PI3KC3 to phagosomes and promotes PI3P production on phagosomes. 2) PI3P mediates the recruitment of NOX2 complex on phagosomes. 3) Rubicon stabilizes NOX2 complex by interacting with p22phox, one of the constituent molecules in NOX2 complex, thereby promotes ROS production within phagosomes. 4) ROS production within phagosomes is hypothesized to recruit ATG8s by unstabilizing the phagosomal membrane (Boyle and Randow, 2015; Martinez et al., 2015).

What is the significance of the role of Rubicon that promotes LAP and LANDO while suppresses autophagy? There is a limit to the lysosomal degradation capacity, and lysosomal overload could lead to lysosomal membrane permeabilization, which causes the release of lysosomal enzymes into the cytosol and activates the cell death pathway. Therefore, it is tempting to speculate that Rubicon might balance the activity of autophagy, phagocytosis and endocytosis, which are independent lysosomal degradation pathways, and in order to avoid lysosomal overload.

#### Microbial Infection and Innate Immune Response

Independent of autophagy, LAP and LANDO, Rubicon is reported to be associated with the exacerbation of microbial infection by suppressing the innate immune response. After viral infection, the expression of Rubicon is increased and Rubicon suppresses interferon response by directly binding to NF-κB essential modulator (NEMO) (Wan et al., 2017; Fang et al., 2019) or interferon regulatory factor 3 (IRF3) (Kim et al., 2017). Rubicon also inhibits cytokine production by binding caspase recruitment domain-containing protein 9 (CARD9). Rubicon-CARD9 complexes disassemble the CBM signaling complex, which consists of CARD9, BCL10, and MALT1, leading to the termination of pattern recognition receptors (PRRs)-induced cytokine production (Yang et al., 2012). As a result, Rubicon permits the intracellular proliferation of virus and fungus, which leads to the aggravation of microbial infection (Yang et al., 2012; Wan et al., 2017). We outlined the autophagy-dependent and–independent role of Rubicon. Whereas Rubicon has various roles, Rubicon exerts its roles properly by changing its binding partners in response to various environmental stimulations. On the other hand, the regulation of these various roles of Rubicon remains largely unknown and future detailed studies are desired.

## Metabolic Diseases and Rubicon

Whereas autophagy has been reported to be protective against various metabolic disease, recent studies have elucidated that autophagy is dysregulated in these settings (reviewed by (Zhang et al., 2018)). In 2016, Tanaka et al. have first elucidated a close relationship between Rubicon and autophagic dysregulation in metabolic disease (Tanaka et al., 2016). In this report, the protein level of Rubicon was increased concomitant with dysregulation of autophagy in the livers of human nonalcoholic fatty liver disease (NAFLD) patients, high fat diet-fed mice, and in the culture hepatocytes treated with saturated fatty acids. Furthermore, they clarified that Rubicon exacerbates hepatic steatosis in high fat diet-fed mice via suppression of autophagy by using hepatocyte-specific Rubicon knockout mice. NAFLD is a major risk factor for hepatocellular carcinoma (HCC), which is increasing worldwide (Huang et al., 2021). Therefore, targeting Rubicon could be a promising approach for therapeutic application in NAFLD, although further analyses are needed whether Rubicon suppression could prevent the development of HCC in NAFLD patients. The precise mechanism that Rubicon suppression ameliorates NAFLD remains unclear. Selective degradation of endoplasmic reticulum (ER-phagy) or lipid droplet (lipophagy) might ameliorate NAFLD as Rubicon suppression relieved ER stress and lipid droplet accumulation in hepatocytes treated with saturated fatty acids (Tanaka et al., 2016). On the other hand, Rubicon suppression might ameliorate NAFLD via selective degradation of nuclear receptor co-repressor 1 (NCoR1), repressor of PPARα, which is recently identified as a selective substrate of autophagy (Saito et al., 2019).

Besides NAFLD, it is reported that there is a close relationship between Rubicon and myocardial infarction (Li et al., 2020; Nah et al., 2020). Nah et al. reported that the expression of Rubicon is up-regulated concomitant with autophagosome accumulation and autophagic cell death (autosis) after myocardial infarction, whereas the suppression of Rubicon ameliorates autophagosome accumulation, and autosis (Nah et al., 2020). On the other hand, Li et al. identified that Rubicon binds to caspase recruitment domain-containing protein 9 (CARD9) during myocardial infarction. They further elucidated that CARD9 deletion exacerbates myocardial infarction via autophagic dysregulation, indicating that autophagic activity is maintained during myocardial infarction via sequestration of Rubicon by CARD9 (Li et al., 2020).

Regarding diabetes mellitus, Aoyama et al. verified the role of Rubicon in pancreatic β cells using pancreatic β cell-specific Rubicon knockout mice, however, Rubicon deletion did not affect glucose homeostasis under a normal diet, and high fat diet (Aoyama et al., 2020). As for diabetic kidney disease, increased Rubicon expression concomitant with autophagic dysregulation was recognized in glomerular podocytes. Notably, upregulation of Rubicon was epidermal growth factor receptor (EGFR) signaling-dependent, and deletion of EGFR in glomerular podocytes ameliorated diabetic kidney disease via Rubicon suppression-dependent autophagic activation (Li et al., 2021). On the other hand, kidney proximal tubules-specific knockout of Rubicon have little influence on the streptozotocin-induced diabetic kidney disease mice model. In addition, kidney proximal tubules-specific Rubicon knockout mice exhibited obesity, hyperlipidemia and NAFLD via the increased exocytosis of fatty acids from kidney proximal tubules (Matsuda et al., 2020). As above, the role of Rubicon in diabetic kidney disease is different for each kidney constituent cells.

## Ageing and Rubicon

Accumulating evidence over the past decade indicates that autophagy has a key role in life span regulation (Hansen et al., 2018; Leidal et al., 2018; Nakamura and Yoshimori, 2018; Wong et al., 2020; Kaushik et al., 2021): autophagy is activated and required for the lifespan extension in various longevity paradigms, whereas autophagic activity is disturbed during the ageing process. On the other hand, the mechanism that autophagic activity decreases during ageing had for a long time been unclear and it became the biggest mystery to be solved in this study field. In 2019, we have elucidated that increment of Rubicon is the leading cause of autophagic disturbance during ageing. Importantly, the increased expression of Rubicon concomitant with autophagic disturbance is highly conserved among species including *Caenorhabditis elegans* (*C. elegans*), *Drosophila* and mammals, and suppression of Rubicon in *C. elegans* promotes longevity via autophagic activation (Nakamura et al., 2019). We also clarified that deletion of Rubicon suppressed organ fibrosis during natural ageing in mice. Furthermore, suppression of Rubicon ameliorated age-related disease, including PolyQ disease model in *Drosophila* and Parkinson’s disease model in mice. The following report has indicated that age-related macular degeneration, one of the representative age-related diseases, could be improved by Rubicon suppression-dependent autophagic activation in retinal pigment epithelial cells (Ando et al., 2021).

Previous reports have indicated that genetic activation of autophagy contributes to lifespan extension in mammals (Pyo et al., 2013; Fernández et al., 2018), however, these studies have forcibly promoted the expression or function of the specific autophagy-related molecules. In contrast, we have for the first time elucidated that lifespan extension could be achieved by the intervention on the molecules that cause physiological autophagic dysregulation during ageing, indicating this study is clinically relevant. Furthermore, this study elucidated that the physical activity was increased by knocking down Rubicon in *C. elegans* and *Drosophila*. Although further analysis using mammals is needed, these results are clinically important because not only lifespan extension but also healthspan extension and sarcopenia prevention are important points in geroscience.

**FIGURE 2:**
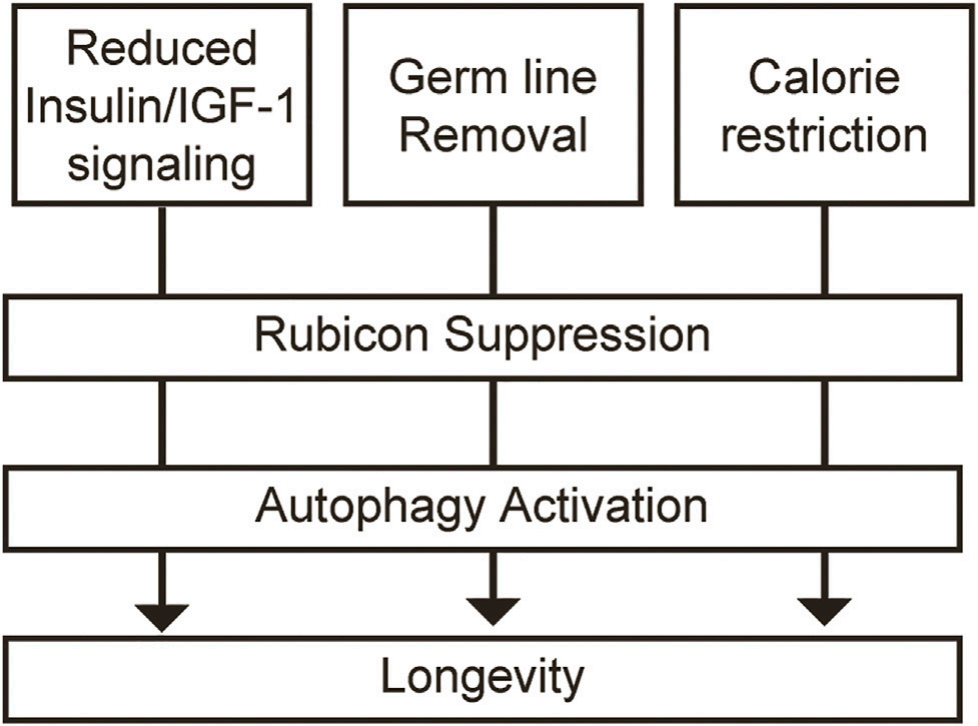
Rubicon repression leads to the longevity *via* activation of autophagy in multiple longevity paradigms. Multiple longevity paradigms, including reduction of insulin/IGF-1 signaling, germ line removal and calorie restriction decrease the expression of Rubicon at the transcriptional level, and leading to the longevity *via* activation of autophagy.

Furthermore, this study elucidated that various longevity paradigms, including *daf-2* mutant (reduction of insulin/IGF-1 signaling), *glp-1* mutant (germ line removal) and *eat-2* mutant (calorie restriction) in *C. elegans*, indicate the decreased expression of *Rubicon* at the transcriptional level, and leading to the longevity via activation of autophagy (Figure 2). Calorie restriction in mice also reduced Rubicon expression, although the precise mechanism remains unclear. Further studies are expected to elucidate the molecular mechanism that various longevity paradigms decrease Rubicon expression. In other words, we should clarify which signaling factor organisms sense and which transcriptional factor convert the signaling factor to increased expression of Rubicon during ageing process. If these questions could be answered, the development of therapeutic strategies against ageing that target autophagy could a reality.

We further investigated which organs of Rubicon are responsible for lifespan extension and clarified that specific knockdown of Rubicon in neuronal cells extended lifespan in *C. elegans* and *Drosophila*. How Rubicon in neuronal cells regulate the ageing process in the whole body? Neuronal cells are known to communicate with peripheral organs via neural circuit as well as humoral factor. Recently, accumulating evidence has elucidated that autophagy-related molecules have an important role in intercellular communication by promoting secretory autophagy (Ponpuak et al., 2015; Padmanabhan and Manjithaya, 2020) and/or exosomal secretion (Xu et al., 2018; Levine and Kroemer, 2019). Therefore, it is tempting to infer that the non-autonomous function of neuronal Rubicon regulates whole body ageing by intercommunicating with the peripheral organ. Future studies are needed to clarify the mechanism that neuronal Rubicon regulates whole body ageing.

Is Rubicon merely a bad guy in age-related disease? We answered the question in 2020 (Yamamuro et al., 2020). This study revealed that suppression of Rubicon in adipose tissue promoted excessive autophagic degradation of PPARγ coactivators, SRC-1 and TIF2, which led to lipodystrophy, insulin resistance, and ectopic fat accumulation including liver steatosis. In other words, Rubicon suppresses excessive autophagic activation and maintains homeostasis in ageing adipose tissue. Interestingly, during ageing process, the expression of Rubicon was significantly suppressed in adipose tissue in contrast to liver and kidney, indicating regulation of Rubicon expression is different for each tissue during ageing. This report is clinically relevant because lipodystrophy, insulin resistance and ectopic fat accumulation, which are closely related to downregulation of Rubicon, are generally observed in the human normal ageing process, and progeria. During ageing, Rubicon is upregulated in the liver and kidney which exacerbate age-related disease including NAFLD and kidney fibrosis, while Rubicon is downregulated in adipose tissue which is also closely associated age-related phenotype including lipodystrophy, insulin resistance and ectopic fat accumulation (Table 1). Elucidating the comprehensive regulatory factor that modulates the tissue-different expression of Rubicon is a future exciting challenge, which could combat the metabolic change of the whole body during ageing.

**TABLE 1:** Differential expression and role of Rubicon in various organs during ageing.

Organ	Age related disease	Rubicon expression by ageing	Ageing phenotype by Rubicon knockout
Liver	NAFLD	Increased	Improved
Kidney	Kidney fibrosis	Increased	Improved
Adipose tissue	Lipodystrophy	Decreased	Deteriorated
	Insulin resistance		
	Ectopic fat accumulation		

A recent report has identified that Rubicon affects male fertility: Rubicon deficiency in Sertoli cells causes defective spermatogenesis via accelerated autophagic degradation of GATA4, which is an essential transcription factor for Sertoli cell function (Yamamuro et al., 2021). This report is interesting from the viewpoint of ageing: it has been previously known that there is a negative correlation between fertility and longevity, although the causal relationship has been unclear (Partridge et al., 2005; Kenyon, 2010). A previous report identified that ELT-5 and ELT-6, which are GATA homologue in *C. elegans* accumulate during ageing and knockdown of ELT-5 and ELT-6 extends lifespan (Budovskaya et al., 2008). Considering together, the regulation of autophagic GATA4 degradation by Rubicon might be one of the mechanisms that explain the negative correlation between fertility and longevity.

The autophagy-independent function of Rubicon has also been identified to contribute to the pathogenesis of age-related disease. Heckmann et al. reported that deficiency of Rubicon-dependent LANDO exacerbates the pathogenesis of Alzheimer disease (Heckmann et al., 2019). Rubicon-dependent LAP is reported to be protective for various age-related disease including age-related macular degeneration (Kim et al., 2013; Muniz-Feliciano et al., 2017). Therefore, we should pay attention to whether the role of Rubicon is autophagy-dependent or not when we consider the function of Rubicon in age-related disease.

## Future Perspectives

Whereas Rubicon inhibition could be a promising therapeutic strategy, several concerns exist. Rubicon inhibition in the whole body might have side effects including lipodystrophy and infertility due to excessive autophagy, and exacerbation of autoimmune diseases, infectious diseases and neurodegenerative disease due to LAP and LANDO inhibition. To overcome these side effects, several issues should be resolved.

First, the regulatory mechanism of Rubicon during ageing should be clarified. Interestingly, there is a close relationship between Rubicon expression and the occurrence of age-related disease: Rubicon expression increases in the liver and kidney during ageing, which leads to the exacerbation of NAFLD, and kidney fibrosis. On the other hand, Rubicon expression decreases in adipose tissue during ageing, which leads to the exacerbation of lipodystrophy, and ectopic fat accumulation (Table 1). Therefore, the identification of the comprehensive regulatory factor of Rubicon during ageing is the inevitable important issue that would enable the development of anti-ageing drug without side effects.

Second, it is critical to identify the region in the brain responsible for lifespan extension by Rubicon suppression. As noted above, Rubicon inhibition in the whole brain might have side effects including Alzheimer disease. Therefore, the site-specific inhibition approach is important to avoid side effects. Recent reports have elucidated that the hypothalamus or neural stem cells regulate ageing (Satoh et al., 2017; Zhang et al., 2017; Kim and Choe, 2019). It is tempting to speculate that Rubicon in these specific cells is essential for general ageing.

Finally, considering the relationship between Rubicon and diseases, human evidence is completely lacking at present. Future studies should accumulate human evidence.

## Conclusion

Rubicon is a cell biologically interesting molecule that plays specific functions: Rubicon is one of few negative regulators of autophagy whereas Rubicon is indispensable for LAP and LANDO. There remain many questions unanswered. What is the regulator of Rubicon during ageing? How Rubicon exerts its various functions properly in response to various situations? Future studies to answer these questions will provide clues to a novel treatment for metabolic diseases and age-related diseases.
